# The Best of Both Worlds: How Combining a Large Language Model and a Rule-Based Algorithm Makes Catheter-Associated Urinary Tract Infection Surveillance More Efficient

**DOI:** 10.1093/cid/ciag301

**Published:** 2026-05-14

**Authors:** Joshua Nordman, Claire Najjuuko, Nicholas Jeschke, Rachael E Snyders, Maria Cristina Vazquez Guillamet, Jordan Shapiro, Carole Leone, Megan Dethloff, Hilary Babcock, Patrick Reich, Kenneth Whalen, Lucy Zhang, Lan Luong, Chenyang Lu, Andrew Atkinson, Jonas Marschall, Abby Sung

**Affiliations:** Division of Infectious Diseases, Washington University School of Medicine, St.Louis, Missouri, USA; Division of Computational and Data Sciences, Washington University in St.Louis, St. Louis, Missouri, USA; Institute for Informatics, Data Science, and Biostatistics, Washington University School of Medicine, St.Louis, Missouri, USA; Division of Infectious Diseases, Washington University School of Medicine, St.Louis, Missouri, USA; Division of Infectious Diseases, Washington University School of Medicine, St.Louis, Missouri, USA; Center for Clinical Excellence, BJC Healthcare, St.Louis, Missouri, USA; Center for Clinical Excellence, BJC Healthcare, St.Louis, Missouri, USA; Center for Clinical Excellence, BJC Healthcare, St.Louis, Missouri, USA; Division of Infectious Diseases, Washington University School of Medicine, St.Louis, Missouri, USA; Center for Clinical Excellence, BJC Healthcare, St.Louis, Missouri, USA; Division of Infectious Diseases, Washington University School of Medicine, St.Louis, Missouri, USA; Center for Clinical Excellence, BJC Healthcare, St.Louis, Missouri, USA; Center for Clinical Excellence, BJC Healthcare, St.Louis, Missouri, USA; Center for Clinical Excellence, BJC Healthcare, St.Louis, Missouri, USA; Department of Computer Science and Engineering, Washington University in St.Louis, St. Louis, Missouri, USA; Division of Infectious Diseases, Washington University School of Medicine, St.Louis, Missouri, USA; Institute for Informatics, Data Science, and Biostatistics, Washington University School of Medicine, St.Louis, Missouri, USA; Division of Infectious Diseases, Washington University School of Medicine, St.Louis, Missouri, USA; Center for Clinical Excellence, BJC Healthcare, St.Louis, Missouri, USA; Division of Infectious Diseases, University of Arizona College of Medicine, Phoenix, Arizona, USA; Division of Infectious Diseases, Washington University School of Medicine, St.Louis, Missouri, USA

**Keywords:** AI, large language model, healthcare-associated infection, catheter-associated urinary tract infection, CAUTI

## Abstract

**Background:**

Catheter-associated urinary tract infection (CAUTI) surveillance is critical for patient safety. Many healthcare systems use electronic algorithms to flag candidate infection events but still require manual chart review to confirm CAUTI cases. We evaluated the use of a large language model (LLM) to enhance CAUTI surveillance.

**Methods:**

We analyzed 919 potential CAUTI cases flagged by the electronic surveillance algorithm at Barnes-Jewish Hospital from 2021 to 2024. These cases were previously classified as 291 CAUTIs and 628 non-CAUTIs by trained infection preventionists (IPs). Several approaches for applying the National Healthcare Safety Network (NHSN) CAUTI definition after extracting clinical data from the electronic medical record (EMR) were compared.

**Results:**

Most patients were female (61.6%) with a median age 68 years (IQR 58, 77). Combining rule-based logic with Clinical Entity Augmented Retrieval (CLEAR) input into an LLM achieved the highest sensitivity (90.0%) and specificity (93.5%). Adjustment of false negatives and false positives after expert adjudication showed sensitivity of 93.6% and specificity of 98.6%. Chart review of false negatives revealed that disagreement with the gold standard mainly occurred due to missing symptom information in clinical documentation provided to the model.

**Conclusions:**

Augmenting the existing algorithmic approach with LLM capabilities significantly enhances CAUTI surveillance and may improve efficiency by reducing the amount of time IPs spend performing manual chart review. Further improvements could be made by optimizing the clinical information presented to the model.

## Teaser

A hybrid approach using a large language model to augment CAUTI event identification by extracting symptoms from clinical documentation achieves 90.0% sensitivity and 93.5% specificity, which shows potential for improving surveillance efficiency.

## Graphical Abstract

**Graphical Abstract:**
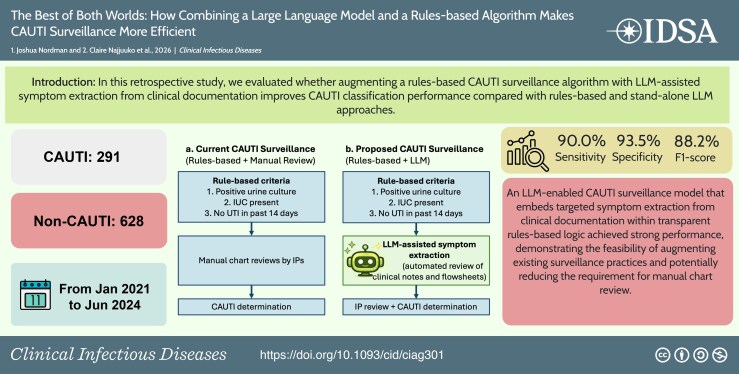
This graphical abstract is also available at Tidbit: https://tidbitapp.io/tidbits/the-best-of-both-worlds-how-combining-a-large-language-model-and-a-rules-based-algorithm-makes-cauti-surveillance-more-efficient-a527e6cb-0f3b-4e10-9a2b-58fa327875c8

**(See the Editorial Commentary by Morgan et al on pages e127–9.)**

Urinary tract infections (UTIs) represent the fifth most frequently reported healthcare-associated infection (HAI), with roughly 62 700 cases documented in United States acute care hospitals in 2015 [1]. The majority (75%) of healthcare-associated UTIs arise from instrumentation of the urinary tract, and catheter-associated UTIs (CAUTIs) are associated with increased morbidity, mortality, and length of stay, thereby placing substantial burdens on patients and healthcare systems [1–3].

The National Healthcare Safety Network (NHSN) provides standardized surveillance definitions that healthcare facilities use to monitor and report CAUTI cases [1]. Many acute care settings continue to rely on manual chart review, which may be supplemented by rule-based electronic decision support systems that combine structured indicators, such as positive urine culture results and presence of an indwelling urinary catheter (IUC), to semi-automate CAUTI surveillance [4–8]. However, the limitations of such surveillance algorithms are well known: they often incorrectly classify CAUTI events because they exclude subjective symptoms, which are not typically stored in structured electronic health record (EHR) data; accordingly, they place a heavy burden on infection preventionists (IPs) and limit operational efficiency [6–10].

Recent approaches have augmented existing electronic surveillance systems with natural language processing (NLP) methods [11, 12]. Advances in NLP, particularly large language models (LLMs), are further reshaping the surveillance landscape by unlocking the potential of free-text clinical documentation. Recent pilot studies highlight the promise of LLMs in HAI surveillance by automating detection workflows [13, 14]. These studies, however, have largely applied LLMs in a standalone manner, fully delegating surveillance decisions to LLMs, and raising concerns about transparency, reliability, and integration into existing workflows.

In this study, we tested a hybrid surveillance paradigm: augmenting traditional rule-based CAUTI surveillance with targeted LLM analysis of clinical notes. In this framework, the LLM is an embedded component within the rule logic, extracting subjective symptoms that complement structured data elements. We hypothesize that this hybrid system will identify potential CAUTIs with improved specificity, while maintaining high sensitivity. By integrating structured rule logic with LLM-enabled subjective symptom extraction, our framework aims to enhance CAUTI surveillance and increase efficiency.

## METHODS

### Setting

**Figure 1.**
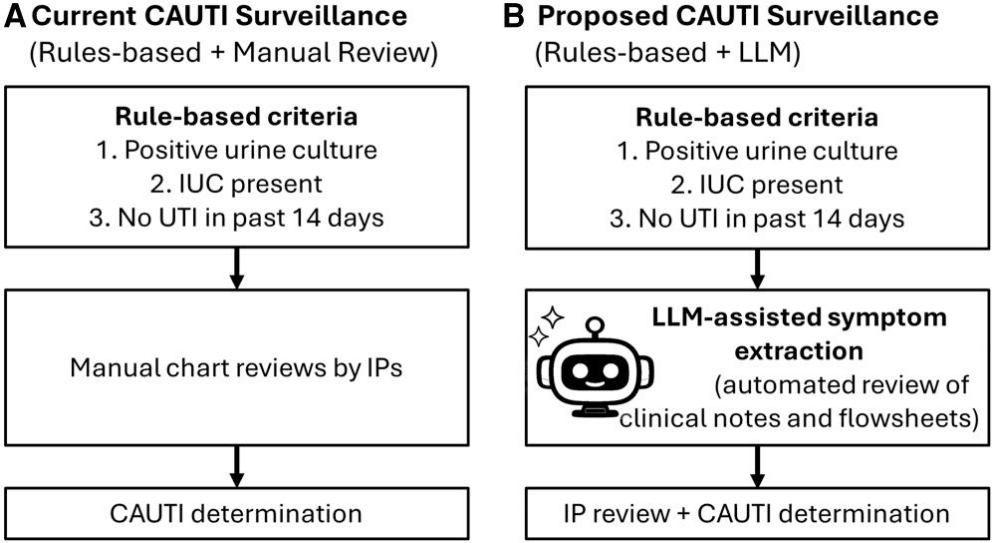
*A*, Existing CAUTI electronic surveillance algorithm with decision support. *B*, Proposed CAUTI surveillance with LLM-assisted symptom extraction. Abbreviations: CAUTI, catheter-associated urinary tract infection; IUC, indwelling urinary catheter; LLM, large language model.

This study used EHR data for all patients flagged as potential CAUTIs by an electronic surveillance algorithm at Barnes-Jewish Hospital (BJH), a 1260-bed academic referral hospital in St. Louis, Missouri, from January 2021 through June 2024. The existing CAUTI surveillance algorithm employs a rule-based approach which includes having a positive urine culture during hospitalization, presence of an IUC, and verification that a given patient did not have a previously confirmed UTI within the preceding 14-day period [6] (Figure 1*A*). This is followed by manual EHR review by trained IPs to verify whether NHSN CAUTI criteria are met.

### Data and Data Processing

Input data included records extracted from Epic (Verona, WI) EHR flowsheet tables, IUC placement and removal dates, clinical notes, and urine culture results from Bugsy—a sub-module in Epic with full chart review capabilities used by IPs for CAUTI surveillance. Flowsheet entries with any mention of genitourinary findings and body temperature recordings were considered. Through consultation with IPs, we selected note types where UTI symptoms are most frequently documented: history and physicals, progress notes, consult notes, emergency department notes, and discharge summaries (Supplementary Table 1). All flowsheets and clinical notes within the infection window period (IWP), a 7-day period starting 3 calendar days prior to the date of urine culture collection and ending 3 calendar days after as defined by NHSN, were included.

The gold standard for reference was the CAUTI or non-CAUTI designations made previously by trained IPs. The dataset included 1155 potential CAUTI cases from 1007 patients; only the earliest flagged case per patient was included. Cases were excluded if more than 2 organisms were identified in urine culture or if IUC insertion/removal dates were missing. The final dataset included 919 potential CAUTI cases, of which 291 (31.7%) were confirmed CAUTIs (Supplementary Figure 1).

### National Healthcare Safety Network Catheter-Associated Urinary Tract Infection Definition Implementation

We sought to further automate the existing CAUTI surveillance procedure while implementing the NHSN definition (as of January 2025) [1] for symptomatic UTI for patients > 1 years old (Figure 1*B*; Supplementary Figure 2). Blood culture data to identify catheter-associated asymptomatic bacteremic UTI were not included.

The date of event (DOE) per case is the date when the first element used to meet NHSN CAUTI criteria occurs for the first time within the IWP [1], which is either the date of the first CAUTI sign/symptom or the date of urine culture collection. NHSN-defined CAUTI signs/symptoms are fever (>38.0°C/>100.4°F), suprapubic tenderness, costovertebral angle pain or tenderness, urinary urgency, urinary frequency, and dysuria. Urinary urgency, frequency, and dysuria were not considered when an IUC was in place since the presence of a device can cause these symptoms [1]. Natural language processing was used to extract these signs/symptoms from flowsheets and clinical notes.

IUC placement and removal dates were used to establish the most recent IUC episode prior to, or coinciding with, the DOE. An IUC episode was defined as a period where the patient had an IUC in place on consecutive calendar days and did not go a full calendar day without an IUC. The start date of this most recent IUC episode prior to the DOE was considered the qualifying IUC placement date. UTI-IUC association was established by checking if the qualifying IUC had been in place for more than 2 consecutive days on the DOE, and was either still present on the DOE or if it was removed, that it had been removed the day before the DOE. If these criteria were not met, the case was classified as non-CAUTI. If UTI-IUC association was established, then cases with signs/symptoms in the IWP were classified as CAUTI and those without signs/symptoms as non-CAUTI.

### Catheter-Associated Urinary Tract Infection Symptom Extraction

A key input for the algorithm described above are CAUTI signs/symptoms stored in flowsheets (including body temperature records and smart-text describing subjective CAUTI symptoms) and clinical notes (including free-text of longer clinical narratives).

#### Rules Regarding Natural Language Processing for Symptom Extraction

This involved using rules to extract signs/symptoms from flowsheets. Fever was identified when recorded body temperature exceeded 38.0°C/100.4°F. Subjective symptoms were extracted using a key-phrase search, considering entries with exact matches to key phrases as valid symptoms (Supplementary Table 2).

#### Large Language Models for Symptoms Extraction

Due to the narrative nature of clinical notes, simple rules-NLP falls short of capturing important context to determine symptom presence. Rules-NLP fails on negation, temporality, uncertainty, and synonyms/abbreviations. Large language models address this by reasoning over longer text spans to produce more accurate labels. A HIPAA-compliant instance of GPT-4o-128k LLM (OpenAI, San Francisco, CA), accessed through Washington University in St. Louis, was used to extract CAUTI symptoms from clinical notes. Three approaches were considered:

- *Naïve prompting*: The full clinical note was provided to the LLM and prompted with the question: “Does the patient have any of the following signs/symptoms on {date}?”
- *Clinical Entity Augmented Retrieval (CLEAR)* [15]: CLEAR was used to extract relevant text excerpts from a full clinical note. The excerpts were used as input into LLM to determine symptom presence. CLEAR included (1) *entity recognition* which identified all clinical entities within a clinical note using BIOMed_NER—a named entity recognition model for biomedical entities [16]; (2) *entity selection* which selected entities most similar to target symptoms, by embedding entities and target symptoms using BioClinicalBERT [17] and retaining entities with a cosine similarity ≥ 0.85 to the target symptom—a threshold chosen to balance relevance and exclusion of irrelevant entities and consistent with the original article describing CLEAR [15]; (3) *entity augmentation*: the filtered entity list was augmented using synonyms from the Unified Medical Language System ontology [18, 19] and manually curated keywords and key phrases; and (4) *chunk retrieval*: entities were identified within the clinical notes, and excerpts of ±150 words before and after these entities were extracted using string similarity matching. Retrieved excerpts were used as LLM input.
- *Retrieval augmented generation (RAG)*: RAG was used to extract relevant text excerpts from a full clinical note based on semantic similarity to target symptoms. RAG included splitting each clinical note into non-overlapping 200-word chunks via sentence-aware segmentation; encoding each chunk into vector embeddings with a biomedical sentence transformer [20]; and storing into Chroma [21], a vector database optimized for similarity search. For each target symptom, the top-*k* (=5) most similar chunks from a clinical note's index were retrieved. Retrieved chunks were de-duplicated and re-ranked with a cross-encoder [22], such that the most semantically similar excerpts to the target symptom appear first when prompting the LLM.

#### Integrating Symptom Sources for Catheter-Associated Urinary Tract Infection Classification

Final CAUTI classification approaches using different methods were compared: (1) *Rules-for-fever-alone* incorporated temperature data from flowsheets; (2) *Rules-NLP* combined flowsheet-derived fever and subjective symptoms from key-phrase matches; (3) *Hybrid (Rules + Naïve LLM Prompting)* combined flowsheet symptoms with clinical notes-derived symptoms obtained using naïve prompting of LLM; (4) *Hybrid-CLEAR* combined flowsheet symptoms with clinical notes-derived symptoms obtained using the CLEAR retrieval pipeline; and (5) *Hybrid-RAG* combined flowsheet symptoms with clinical notes-derived subjective symptoms obtained using the RAG pipeline. These configurations allowed us to quantify the incremental value of LLM-assisted extraction relative to flowsheet-only rule-based approaches. In another approach, (6) *LLM for CAUTI classification*, CAUTI determination was fully delegated to the LLM via chain-of-thought decision-logic prompting. For each patient, a text-based summary containing the urine culture collection date and organisms, a time-ordered symptom history, and IUC placement/removal timeline was constructed. The LLM was prompted with an explicit, step-by-step rendering of the NHSN CAUTI definition and asked to apply the criteria to the patient summary and return a CAUTI classification (Supplementary Table 3). Catheter-associated urinary tract infection classification for each approach was evaluated using sensitivity, specificity, precision, and F1-score.

### Ethical Considerations

IRB approval was obtained through the Washington University in St. Louis Human Research Protection Office. All patient data was de-identified to maintain patient confidentiality and comply with legal standards.

## RESULTS

Table 1 provides patient demographics and hospital stay information. Table 2 compares 6 CAUTI classification algorithms evaluated against the gold standard. Among 919 catheterized patients with positive urine cultures with a bacterial quantification of ≥10^5^ CFU/mL, the Hybrid-CLEAR model had the highest sensitivity (90%). Using fever alone, the tool identified 77.7% of all CAUTI cases. Including subjective symptoms from flowsheets, the tool identified 80.4% of all CAUTI cases, compared to 90.0% when subjective symptoms from flowsheets and clinical notes were included via Hybrid-CLEAR. Systematically extracting the most relevant text before querying the LLM for subjective symptoms resulted in improved sensitivity compared to other symptom extraction methods.

**Table 1.** Patient Characteristics

#	Variable	All (N = 919)	CAUTI (n = 291)	Non-CAUTI (n = 628)
1	Sex (n, %)			
	Male	353 (38.4)	119 (40.9)	234 (37.3)
	Female	566 (61.6)	172 (59.1)	394 (62.7)
2	Age, years, median (IQR)	68 (58, 77)	67 (54, 75)	69 (59, 77)
3	Length of hospital stay, days, median (IQR)	20 (12, 38)	23 (14, 40)	19 (11, 35)
4	Dwell time before urine culture, days, median (IQR)	NA	6 (3, 10)	NA
5	Time between admission date and urine culture collection, days, median (IQR)	10 (5, 19)	11 (6, 22)	10 (5, 18)

Abbreviations: CAUTI, catheter-associated urinary tract infection; IQR, interquartile range.

**Table 2.** Performance of Different Methods

#	Method	Sensitivity	Specificity	F1-score
1	Rules-fever alone	77.7%	97.1%	84.5%
2	Rules-NLP	80.4%	95.2%	84.3%
3	Hybrid (Rules + Naïve LLM Prompting)	88.7%	86.5%	81.4%
4	Hybrid-CLEAR	90.0%	93.5%	88.2%
5	Hybrid-CLEAR (modified)	93.3%	98.5%	95.1%
6	Hybrid-RAG	83.9%	93.8%	85.0%
7	LLM alone	88.7%	86.3%	81.3%

Abbreviations: CLEAR, Clinical Entity Augmented Retrieval; LLM, large language model; NLP, natural language processing; RAG, retrieval augmented generation.

After review, a significant proportion of Hybrid-CLEAR's false negatives or false positives compared to the gold standard were found to represent true negatives or positives by NHSN criteria: 32 cases characterized as false positives were true positives, and 8 false negatives were true negatives (Tables 3 and 4). Modified sensitivity and specificity adjusting for these discrepancies (Table 2) show improvement in the model's sensitivity (93.3%), specificity (98.5%), and F1-score (95.1%).

**Table 3.** Reasons for Hybrid-CLEAR False Negatives

	False Negatives (29/291)	
#	Reason	n (%)
1	Symptoms identified by IPs, but not present in clinical notes and flowsheets available in model development	15 (51.7)
2	Does not satisfy NHSN CAUTI definition for catheter association	7 (24.1)
3	IUC in place on date of identified symptom but not in place at time symptom was noted^a^	6 (20.7)
4	Does not satisfy NHSN CAUTI definition of all CAUTI criterion being within the IWP	1 (3.5)

Abbreviations: CAUTI, catheter-associated urinary tract infection; IPs, infection preventionists; IUC, indwelling urinary catheter; LLM, large language model; NHSN, National Healthcare Safety Network.

^a^Exact timing information was not considered in model development.

**Table 4.** Reasons for Hybrid-CLEAR False Positives

	False Positives (41/628)	
#	Reason	n (%)
1	Satisfies NHSN CAUTI definition	32 (78.1)
2	LLM misattribution/misclassification of symptoms^a^	7 (17.1)
3	Urine culture grew <1000 CFU/mL. This case should not have been a candidate CAUTI if filtered appropriately	1 (2.4)
4	Present on admission—symptomatic UTI	1 (2.4)

Abbreviations: CAUTI, catheter-associated urinary tract infection; LLM, large language model; NHSN, National Healthcare Safety Network; UTI, urinary tract infection.

^a^Symptoms from a non-CAUTI etiology were linked to CAUTI (eg, low back pain from a vertebral fracture; flank pain from myositis; large urine output from diuresis as urinary urgency).

Among 29 false negatives, more than half (51.7%) had symptoms identified by IPs but were not present in the clinical documentation used for model development. This could be attributable to symptoms being documented in note types not included in the dataset, or discrepancies between what was documented and what was interpreted by IPs. Additionally, 6 cases had IUCs present on the symptom date but removed before the exact time of symptom documentation, an exact timing factor not considered in our model.

Among 41 false positives, 7 were due to LLM misattribution of symptoms, where symptoms from non-CAUTI etiologies were incorrectly linked to CAUTI (eg, low back pain from a vertebral fracture, flank pain from myositis, or urinary urgency due to medication-induced diuresis). Additionally, one case had urine culture growth of <1000 CFU/mL, which should have been excluded by the existing electronic filter. Another case was a present-on-admission UTI.

## DISCUSSION

Performing CAUTI surveillance requires significant effort from healthcare systems and IPs. Surveillance activities require an estimated one-quarter to one-half of personnel work hours [23], and IPs at our institution dedicate up to 6 hours/week on CAUTI surveillance, with the entire team spending up to 60 hours/week on HAI surveillance. To improve efficiency, electronic surveillance algorithms integrating microbiology data and IUC utilization have been developed to flag potential CAUTIs. Other groups have tested algorithms that employed these structured data alone, and while they achieved 100% sensitivity, specificity was as low as 2.8%, indicating excessive false positives in the absence of subjective clinical information such as symptoms. In order to obtain this information and maximize performance, substantial human review is still required [24]. Recent advances in LLMs now offer the opportunity to process these unstructured data and reduce the manual chart review requirement.

Our Hybrid-CLEAR model builds on these advances by combining structured, rule-based logic with LLM-enabled symptom extraction. This approach builds on RAG, a foundational approach wherein relevant text excerpts are retrieved from clinical notes before being passed to the LLM [25–27]. By restricting the LLM's input to high-yield information, RAG performs better than providing full notes, which can suffer from information overload and a “lost in the middle phenomenon,” wherein data in the middle of inputted sets are at risk of being inadequately evaluated [28]. The Hybrid-CLEAR model adapts the CLEAR framework which enhances RAG through entity-based retrieval of relevant text excerpts [15]. CLEAR ensures that the LLM focuses only on relevant clinical information, leading to more accurate symptom extraction and improved performance.

When compared with the current surveillance process, the Hybrid-CLEAR model demonstrated good performance, with 90.0% sensitivity and 93.5% specificity. It outperformed a rule-based algorithm with fever alone (sensitivity: 77.7%), or rules-NLP where NLP was used to include subjective symptoms from smart-text (sensitivity: 80.4%). Furthermore, Hybrid-CLEAR outperformed standalone use of LLMs (sensitivity: 88.7%). Comparisons between models are descriptive, and small differences in performance may not be statistically significant.

Although 90% sensitivity is not adequate for surveillance purposes, this model identified cases incorrectly classified by existing surveillance practices. Seventy-eight percent of its false positives were valid CAUTIs as defined by NHSN criteria, reflecting cases detected by our model but missed by current review methods. Similarly, 27.6% of false negatives did not meet NHSN criteria despite having been previously reported as CAUTIs by IPs. An adjusted evaluation incorporating these discrepancies shows improved sensitivity: 93.3% and specificity: 98.5%. Alshanqeeti et al noted similar improved metrics after expert review of GenAI output [29], a result that reflects the inherent subjectivity and variability in expert adjudication and could signal broad performance improvement in the future with implementation of this paradigm. As these technologies are incorporated into healthcare workflows, human supervision may take on a slightly different role, as much of the time-consuming and error-prone chart review could be relegated to AI, while a final “quality control” step by a human remains necessary for validation.

Automatically integrating subjective clinical information with objective algorithms can improve efficiency by reducing chart review time by summarizing cases before review and focusing IPs’ attention to areas in the chart where NHSN CAUTI criteria have been identified by the model. In contrast to fully automating CAUTI detection using LLMs alone [13, 14], our proposed use of the hybrid framework is to preserve the essential IP surveillance workflow to ensure LLMs augment rather than replace expert judgment. This approach supports prior cautions against fully automating complex surveillance procedures due to data incompleteness, ambiguity, and need for contextual judgment [30].

Our model has implications for operational efficiency beyond accuracy. From 2021 to 2024, the electronic surveillance system at BJH identified 919 potential CAUTI cases for review by IPs. Under the Hybrid-CLEAR model, restricting review to only the 303 cases classified as positive would have reduced the review workload by approximately 66%, a substantial decrease in IP time dedicated to CAUTI review. This efficiency gain is critical because it would free up time for trained IPs to redirect their efforts toward more impactful prevention strategies, quality improvement, and educational initiatives [31, 32].

Despite these promising results, there are several limitations. Expert review of the model's false negatives showed that most (51.7%) were due to symptoms identified by IPs that were missing in the clinical documentation presented to the model. Similarly, a recent study using LLMs to detect central line-associated bloodstream infections found that limited data access was a significant source of LLM errors [31]. This reflects the importance of optimizing the information presented to LLMs. Second, 17.1% of false positives were due to LLM misattribution of symptoms, where symptoms from non-CAUTI etiologies were incorrectly linked to CAUTI. This obliges further LLM prompt refinement. Third, there is a modest level of complexity related to NHSN rules, and while our model implemented these criteria faithfully, complicated cases (20.7% of false negatives) related to catheter timing and symptoms documentation were misclassified. Fourth, there are cost considerations that may limit the uptake of LLM-enhanced surveillance (Supplementary Table 4). Fifth, this model was trained on charts from a single healthcare system, so performance may differ at other institutions with different note and writing styles as well as the documentation of structured data. Additionally, developing and maintaining LLM-enhanced systems requires technical expertise and ongoing model updates, which may offset some efficiency gains if not supported by institutional infrastructure.

In summary, a hybrid model combining rule-based algorithms with LLM-enabled symptom extraction enhanced performance of existing CAUTI surveillance methods. By maintaining the essential role of expert review while reducing time burden through intelligent case prioritization, this approach promises to increase surveillance efficiency without fundamentally changing IP workflow. As this was done in an Epic environment, it could be possible to incorporate future iterations into Epic's IP module. Due to variations in how institutions perform surveillance, this framework should be replicated and expanded upon to ensure results are consistent, as it has significant implications for how surveillance could be performed in the future. Implementation efforts should prioritize optimization of clinical information presented to the model and further improvements in LLM prompting. Furthermore, the methodological framework demonstrated here—particularly the integration of CLEAR information retrieval for LLM enhancement with rule-based algorithms—may extend to surveillance of other HAIs including surgical site infections and central line-associated bloodstream infections, where subjective documentation and multimodal data similarly play a central role. Such expansion could broadly enhance HAI surveillance efficiency and free up specialist time for prevention and education work that may have greater impact on patient outcomes.

## Supplementary Material

ciag301_Supplementary_Data

## Supplementary Data

Supplementary materials are available at *Clinical Infectious Diseases* online. Consisting of data provided by the authors to benefit the reader, the posted materials are not copyedited and are the sole responsibility of the authors, so questions or comments should be addressed to the corresponding author.
