# Supplementary material for: The Best of Both Worlds: How Combining a Large Language Model and a Rule-Based Algorithm Makes Catheter-Associated Urinary Tract Infection Surveillance More Efficient
Source: Clin Infect Dis. 2026 May 14;83(1):e120–6. doi: 10.1093/cid/ciag301 (PMC13393108; doi:10.1093/cid/ciag301)
Supplement: ciag301_Supplementary_Data [file ciag301_supplementary_data.pdf]

Supplement
The Best of Both Worlds: How Combining a Large Language Model and a Rules-based Algorithm Makes CAUTI Surveillance More Efficient

<u>Supplementary Figures</u>

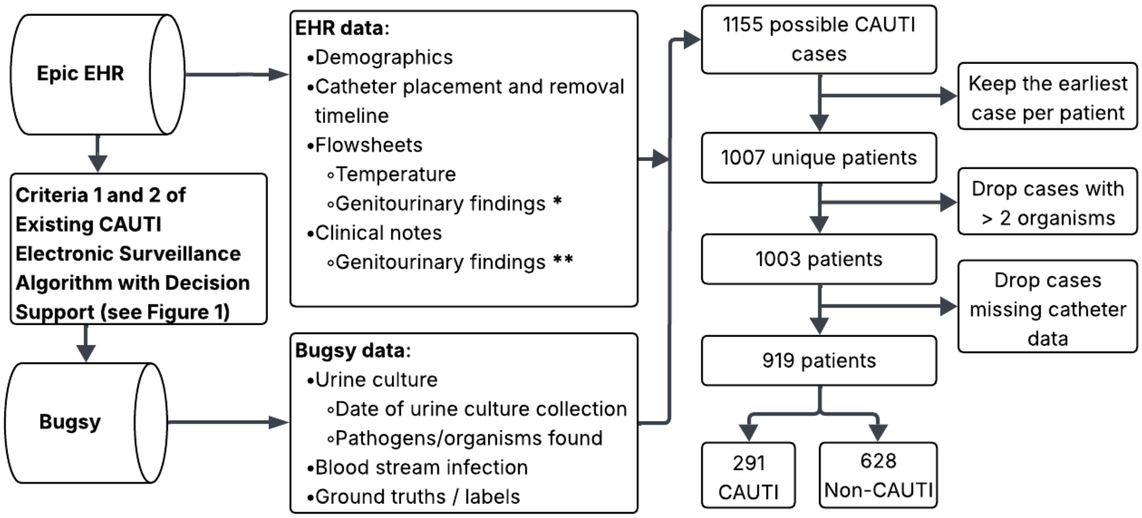

Supplement Figure 1: *Data sources and study population. All data is structured except for items with a single asterisk (smart text) or double asterisk (free text). CAUTI - Catheter Associated Urinary Tract Infection; EHR - Electronic Health Records.*

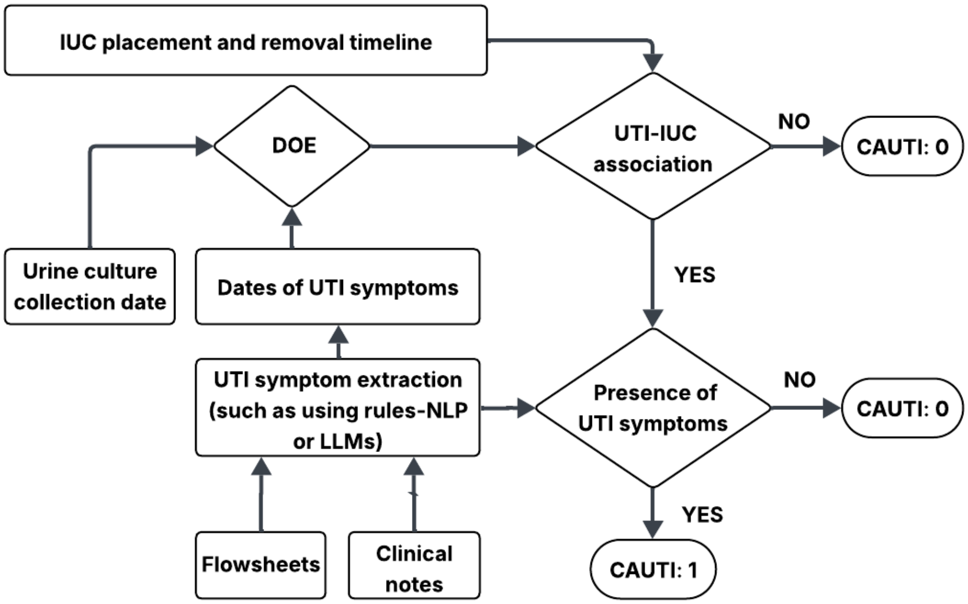

Supplement Figure 2: *CAUTI Identification Procedure. CAUTI– Catheter Associated Urinary Tract Infection; DOE – Date of Event; IUC - Indwelling Urinary Catheter; LDA – Lines Drainages and Airways; UTI – Urinary Tract Infection.*

Supplementary Tables

Supplement Table 1: All note types and selected note types

#	All Note Types	Selected Note Types
1	ACP (Advance Care Planning)	No
2	Assessment & Plan Note	Yes
3	Brief Op Note	No
4	Consults	Yes
5	Consults, Subsequent	Yes
6	Discharge Instructions	No
7	Discharge Summary	Yes
8	ED Procedure Note	No
9	ED Provider Notes	Yes
10	ED Re-evaluation Note	No
11	H&P	Yes
12	Hospital Course	Yes
13	Incidental Note	No
14	Interval H&P Note	Yes
15	Miscellaneous	No
16	Operative Note	No
17	OR Surgeon	No
18	Plan of Care	No
19	Post-Procedure Note	No
20	Pre-Cardiac Catheterization Workup and H&P	No
21	Pre-Procedure Note	No
22	Pre-Sedation Documentation	No
23	Procedures	No
24	Progress Notes	Yes
25	Result Encounter Note	No
26	Significant Event	No
27	Subjective & Objective	No
28	Subsequent	Yes
29	Summary of Treatment Recommendations Non-Billable	No
30	Telephone Encounter	No
31	Treatment Plan	No

Supplement Table 2: Key-phrases list used for searching CAUTI symptoms from flowsheets

#	Symptom	Key-phrases list
1	Suprapubic tenderness	'suprapubic tenderness', 'suprapubic', 'bladder pain', 'pelvic discomfort', 'pelvic pain', 'lower abdominal pain',
2	Costovertebral angle pain or tenderness	'costovertebral angle pain or tenderness', 'costovertebral angle pain', 'CVA tenderness', 'flank pain', 'lower back pain', 'low back pain',
3	Urinary urgency	'urinary urgency', 'urgency',
4	Urinary frequency	'urinary frequency', 'excessive passage of urine', 'excessive urination', 'excessive urination at night', 'increase urinary output',
5	Dysuria	'dysuria', 'pain with urination', 'painful urination',

Supplement Table 3: Large Language Model Prompts

#	Prompt
1.	**Naïve Prompt**<br><br>""""<br>Your role is to review clinical notes to extract symptoms of urinary tract infections. Now analyze the following clinical note:<br><br>***"{note text}"***<br><br>Does patient have any of the following signs or symptoms reported on ***{date}*****:**<br>• suprapubic tenderness: (whether elicited by palpation (tenderness-sign) or provided as a subjective complaint of suprapubic pain (pain-symptom). Lower abdominal pain or bladder or pelvic discomfort are examples of symptoms that can be used as suprapubic tenderness. Generalized "abdominal pain" in the medical record is too general and not to be interpreted as suprapubic tenderness as there are many causes of abdominal pain.)<br>• costovertebral angle pain or tenderness: (Lower back pain (left, right, or bilateral) or flank pain (left, right, or bilateral) are examples of symptoms that can be used as costovertebral angle pain or tenderness. Generalized "low back pain" is not to be interpreted as costovertebral angle pain or tenderness)<br>• urinary urgency (An abrupt, strong, often overwhelming, need to urinate.)<br>• urinary frequency (Abnormally frequent urination.)<br>• dysuria (Pain or a burning sensation when urinating.)<br><br>If the note mentions the symptom occurred another date rather than ***{date}*****,** (e.g., "yesterday", or on a specific earlier date), infer the actual date of occurrence.<br>""""
2.	**Prompt for Hybrid, CLEAR, and RAG pipelines**<br><br>""""<br>Your role is to review clinical notes to extract symptoms of urinary tract infections. Now analyze the following:<br><br>Clinical notes: ***"{note text}"***<br><br>Does the patient have symptom *****{symptom}***** also known as ***{symptom_aka}*** noted on ***{date}?***

	If the {symptom} is mentioned but occurred on another date rather than ***{date}***, (e.g., "yesterday", or another past date), infer and return the actual date of occurrence.<br>"""
**3.**	**Chain-of-thought prompt: step-by-step rendering of the NHSN CAUTI definition**<br><br>"""<br>Patient Description: ***{patient_text}***<br><br>Your task is to determine if the patient described above has a Catheter-Associated Urinary Tract Infection (CAUTI) according to the National Healthcare Safety Network (NHSN) guidelines. Analyze the patient information, apply the CAUTI criteria, and classify the case with reasoned determination.<br><br>Here is the NHSN CAUTI Decision Algorithm<br><br>**START**<br><br>**Step 1:** Positive urine culture with no more than two species of organisms, at least one of which is a bacterium of >10^5 CFU/ml.<br>• No: STOP. Does not meet UTI criteria<br>• Yes: Go to Step 2<br><br>**Step 2:** Had an indwelling urinary catheter that had been in place for more than two days on the date of event AND was either: Still present for any portion of the calendar day on date of event, OR Removed day before date of event?<br><br>Please Note: Indwelling urethral catheters (IUCs) that are removed and reinserted: If, after an IUC removal, the patient is without an IUC for at least 1 full calendar day (NOT to be read as 24 hours), then the IUC day count will start anew. If instead, a new IUC is inserted before a full calendar day has passed (for example removed on December 4 and reinserted December 5), the IUC device day count, to determine eligibility for a CAUTI, will continue uninterrupted.<br>• No: STOP. Does not meet CAUTI criteria<br>• Yes: Go to Step 3<br><br>**Step 3:** At least one of the following signs or symptoms during the infection window period? Fever (>38.0 (°C) or >100.4 (°F)), suprapubic tenderness, costovertebral angle pain or tenderness, urinary urgency, urinary frequency, dysuria.<br>Please Note: Urinary urgency, urinary frequency, and dysuria symptoms cannot be used when catheter is in place.<br>• Yes: Meets criteria for catheter associated SUTI (CAUTI). STOP<br>• No: Go to Step 4<br><br>**Step 4:** Organism identified from blood specimen with at least one matching bacterium to bacterium in the urine at ≥ 100,000 CFU/ml?<br>• Yes: Meets criteria for catheter associated ABUTI (CAUTI). STOP<br>• No: Does not meet UTI criteria<br><br>**END**<br>"""

Supplement Table 4: Time and Cost Analysis

Method	Time per case	Cost per case
Rules-Fever alone	~0.0018 mins	$0
Rules-NLP	~ 0.0018 mins	$0
Hybrid (Rules + Naïve LLM Prompting)	~0.27 mins	$0.10
Hybrid-CLEAR	~ 1.14 mins	$0.20
Hybrid-RAG	~ 0.89 mins	$0.15
LLM alone	~1.20 mins	$0.21

We evaluated runtime and costs across all CAUTI classification methods. Rule-based approaches (Rules-Fever alone and Rules-NLP) were fastest, requiring ~0.0018 minutes per case with no costs since they used only structured data without LLM calls.

LLM-enhanced methods showed higher runtime requirements. The Hybrid (Rules + Naïve LLM Prompting) was fastest among LLM methods (0.27 minutes, $0.10 per case), while the standalone LLM approach required the longest runtime (1.20 minutes, $0.21 per case). The Hybrid-CLEAR (1.14 minutes, $0.20 per case) and Hybrid-RAG (0.89 minutes, $0.15 per case) showed intermediate runtime.

All costs reflect per-token API charges for OpenAI's GPT-4o-128k LLM, with variable per-case costs depending on clinical note length. Despite runtime differences, all methods completed processing within 1.5 minutes per case, making them feasible for real-time clinical implementation.

The Hybrid-CLEAR model used an average of 1.14 minutes at a cost of $0.20 to process each case. All classification approaches required under 1.5 minutes per case, representing a negligible time requirement for practical implementation. The efficient processing time suggests that our method is highly feasible for real-time automated CAUTI surveillance systems. The cost reflects API access charges for OpenAI's GPT-4o-128k LLM, which is billed per token. This implies variable per-case costs depending on the volume of text in each patient's clinical records.
